# Systematic review and meta-analysis: the advantage of endoscopic Mayo score 0 over 1 in patients with ulcerative colitis

**DOI:** 10.1186/s12876-022-02157-5

**Published:** 2022-03-03

**Authors:** Angelo Viscido, Marco Valvano, Gianpiero Stefanelli, Annalisa Capannolo, Chiara Castellini, Eugenia Onori, Antonio Ciccone, Filippo Vernia, Giovanni Latella

**Affiliations:** 1 Gastroenterology Unit, Division of Gastroenterology, Hepatology, and Nutrition, Department of Life, Health and Environmental Sciences, University of L’Aquila, Piazzale Salvatore Tommasi 1, 67100 L’Aquila, Italy; 2 Andrology Unit, Department of Life, Health and Environmental Sciences, University of L’Aquila, L’Aquila, Italy; 3 Internal Medicine and Nephrology Unit, Department of Life, Health and Environmental Sciences, University of L’Aquila, L’Aquila, Italy; 4 Gastroenterology and Digestive Endoscopy Unit, ASUR 1, Pesaro, Italy

**Keywords:** Ulcerative colitis (UC), Mayo endoscopic score (MES), Mucosal healing (MH), Steroid-free clinical remission, Inflammatory bowel disease (IBD)

## Abstract

**Background:**

Mucosal healing (MH) evaluated by endoscopy is a novel target of therapy in UC as it is associated with improved long-term outcomes. It is defined based on the Mayo endoscopic score (MES), but it is still to define whether a value of MES 0 or 1 should be the target. The purpose of this paper is to present the results of a systematic review with meta-analysis which compares long-term outcomes of patients in steroid-free clinical remission with MES 0 with those with MES 1.

**Methods:**

A systematic electronic search of the literature was performed using Medline, Scopus, and CENTRAL through December 2020 (PROSPERO n:CRD42020179333). The studies concerned UC patients, in steroid-free clinical remission, with MES of 0 or 1, and with at least 12-months of follow-up.

**Results:**

Out of 4611 citations, 15 eligible studies were identified. Increases in clinical relapse among patients with MES 1 were observed in all the studies included in this review, suggesting that MES of 1 have a higher risk of relapse than a score of 0. MES 0 patients displayed a lower risk of clinical relapse (OR 0.33; 95% CI 0.26–0.43; I^2^ 13%) irrespective of the follow-up time (12-months or longer). On the other hand, no differences were found comparing MES 0 versus MES 1 about the risk of hospitalization or colectomy.

**Conclusions:**

MES 0 is associated with a lower rate of clinical relapse than is MES 1. For this reason, MES 0, rather than MES 0–1, should be considered the therapeutic target for patients with UC.

**Supplementary Information:**

The online version contains supplementary material available at 10.1186/s12876-022-02157-5.

## Background

Ulcerative colitis (UC) is an idiopathic inflammatory bowel disease that generally begins in young adulthood and lasts a lifetime with a chronic relapsing course [1]. The incidence is increasing worldwide, with no definitive cure as yet available. Although the exact etiology of UC remains unknown, the pathogenesis of the colonic inflammatory lesions appears to be due to a dysregulation of the gut mucosa immune system. Medical therapy has been directed to correcting this immunologic imbalance [1–4].

Inflammation of the colonic mucosa is responsible for signs, symptoms, and complications of UC. Both the development of complications and refractoriness to medical therapy may lead to the requirement for colectomy [3–5].

The clinical presentation usually depends on the severity (activity) and the extent of the colonic lesions, although the correlation between symptoms and lesions is not a rule. Patients with UC may lack, or display only minimal, symptoms despite the evidence of active and extensive inflammation at colonoscopy [1, 2]; and symptoms may be present, despite the absence of inflammatory activity at endoscopy [6, 7], which could be related to concomitant irritable bowel syndrome [8, 9].

Resolution of symptoms and maintenance of clinical remission have traditionally been considered as the best targets of therapy in UC [1–4]. Such strategy, however, does not appear to have significantly changed the natural course of the disease [10, 11]. In the last few years, the target of therapy has moved from the resolution of symptoms to the healing of colonic mucosal lesions [3, 4, 12]. Mucosal healing (MH), compared with clinical remission, is associated with a lower risk of recurrence and complications at follow-up [13–16]. Thus, a strategy that aims at MH could allow us to change the natural history of UC.

Current guidelines indicate that endoscopic evaluation represents a necessary tool to define remission and to make clinical decisions [3, 4]. To date, however, no consensus exists regarding the definition of endoscopic remission. Various score systems and cut-off values have been proposed, but the most appropriate one has yet to be established [13–16]. The Mayo endoscopic score (MES) is the most commonly adopted score to measure endoscopic activity in both clinical trials and daily practice [17, 18]. Endoscopic remission is usually defined as either normal mucosa (MES 0) or mild erythema and mild friability (MES 1) appearance [13, 15]. Agrowing number of observations point to the association ofMH with improved long-term clinical outcomes [16, 19].

To identify the best definition of endoscopic remission using the MES, we performed a systematic review and meta-analysis to compare MES 0 versus MES 1 in patients with UC in steroid-free clinical remission. Clinical relapse, hospitalization, and colectomy were considered as outcomes to evaluate the changes in the clinical course.

## Methods

### Study protocol

Our systematic review and meta-analysis were conducted according to PRISMA guidelines [20]. We used a predetermined protocol (PROSPERO n: CRD42020179333; submitted in April 2020).

A systematic electronic search of the literature was performed using PubMed/MEDLINE, Scopus, and CENTRAL. The search included a combination of Medical Subject headings (MeSH) and keywords (Additional file 1: Table S1). Each of the relevant publication reference sections, Google Scholar, relevant abstracts from United European Gastroenterology Week, and European Crohn’s and Colitis organization conferences were also screened for other applicable publications. The last search was performed in December 2020.

The Authors of the eligible studies were contacted for additional information regarding any inconsistencies in their reported results.

The search was limited to studies conducted on human, adult UC patients in steroid-free clinical remission with MES 0 or MES 1 at endoscopic evaluation. Studies on pediatric populations (0–18 years) were excluded. We considered non-randomized study of intervention (NRSI): prospective and retrospective cohort studies, and case–control studies.

The datasets generated and analysed during the current study are available in the “Mendeley data” repository (https://data.mendeley.com/). The details to access are: Systematic Review and Meta-Analysis: the advantage of Endoscopic Mayo Score 0 over 1 in patients with Ulcerative Colitis. Published: 1 February 2022; Version 1 DOI https://doi.org/10.17632/rkhsvzwr6v.1

### Definition of Mayo endoscopic 0 or 1

We identified a dichotomist group of patients with MES of 0 or 1. According to the literature, MES 0 score indicating a normal mucosa; MES 1 score indicating a decreased vascular pattern, erythema, and mild friability [21, 22].

### Definition of clinical remission

We included only patients in steroid-free clinical remission. Clinical remission was defined by disease activity scores (e.g., Partial Mayo Score, Truelove and Witts Score, Simple Clinical Colitis Activity Index) below thresholds set in the individual studies [22].

### Definition of clinical outcomes

Our primary analysis was the comparison of the proportion of patients with clinical relapse in the MES 0 vs MES 1 group.

Clinical outcomes evaluated in our study included (a) clinical relapse, (b) IBD-related hospitalization rate, and (c) colectomy. Clinical relapse was defined by disease activity scores (e.g., Partial Mayo Score, Truelove and Witts Score, above-set thresholds determined by individual studies) and/or need for medication intensification (Additional file 1: Table S2).

### Subgroup analysis and sensitive analysis

A subgroup analysis was performed comparing studies with follow-up periods less than 12 months with those greater than 12 months.Moreover, we performed a subgroup analysis of studies including patients on conventional therapy, biological therapy, or both.

Sensitivity analysis was also conducted excluding the abstracts and the studies with low-moderate quality assessed by the New Castle-Ottawa scale (NOS). Moreover, a sensitivity analysis of studies that included only clinical relapse defined by Partial Mayo Score and among the studies with a prospective design was performed.

### Selection of studies

Three authors (MV, AC, and GS) independently reviewed abstracts and manuscripts for eligibility. Conflicts were resolved by consensus with senior authors (AV, GL). Inclusion and exclusion criteria are outlined below.

#### Inclusion criteria

1. Human studies comprisingpatients 18 years or older with known UC;
2. NRSI: prospective and retrospective cohort studies, and case–control studies;
3. at least 12 months of follow-up;
4. steroid-free clinical remission;
5. no prior colectomy;
6. studies on UC patients evaluating the association between mucosal healing assessed in terms of MES 0 or MES 1 and clinical outcomes (clinical relapse, hospitalization, colectomy).

#### Exclusion criteria

1. Non‐Human Studies (cell culture, animal models);
2. pediatric cohorts (patients less than 18 years of age);
3. review articles and other systematic reviews or meta-analyses;
4. absence of steroid-free clinical remission at inclusion;
5. inability to distinguish between MES 0 and MES 1;
6. clinical outcomes not reported.

### Data extraction and assessment of studies quality

Data extraction was carried out independently by two investigators (MV and GS). Any discrepancies were resolved by consensus in consultation with the senior authors (AV and GL).

Each reviewer extracted the following data: title and reference details (first author, journal, year, country), study population characteristics (number of patients included in study, gender and age, MES, IBD-related medications) outcome data (clinical relapse, hospitalization, colectomy). All data were recorded independently by both literature reviewers in separate databases and were compared at the end of the reviewing process to limit selection bias. The database was then reviewed by a third person (AV). Two authors (MV and GS) independently assessed the quality of included studies using the Newcastle‐Ottawa scale (NOS) for case‐control studies or cohort studies [23]. Significant conflicts between NOS scores were resolved by consensus and consultation of senior authors (AV, GL), otherwise, scores were averaged between the two reviewers. Criteria evaluated for assesment of the quality of the included studies are reported in Additional file 1: Table S4. NOS scores were defined as high (score 7–9), moderate (score 4–6), or low (score 0–3).

### Statistical analyses

Adjusted odds ratios (OR) from individual studies were extracted using dichotomous data (MES 0 vs MES 1 groups) for each outcome (clinical relapse, hospitalization, and colectomy). Review Manager v5.4.1 (RevMan 2020, Copenhagen, The Cochrane Collaboration) was used to calculate the pooled odds ratio (95% CI and *p* values) of clinical relapse, hospitalization, and colectomy among UC patients with MES 0 versus MES 1, generate forest plots, calculate the *I*^2^ statistic and to generate a funnel plot. Heterogeneity was assessed using the *I*^2^ statistic defined by the Cochrane Handbook for Systematic Reviews [24]. The Odds Ratio for the individual study was combined using a fixed-effect model, with a random-effects model planned in case of substantial heterogeneity (*p* < 0.10, *I*^*2*^ > 50%). Either Chi^2^ test *p* < 0.10 or *I*^2^ value > 50% indicated substantial heterogeneity. Publication bias was graphically determined using funnel plots: a symmetric inverted funnel shape arises from a ‘well-behaved’ data set, in which case the publication bias is unlikely [25]. Funnel plot asymmetry was explored using Egger's test [26]. The extracted data were analyzed using the R statistical software (version 3.0.3; R Foundation for Statistical Computing, Vienna, Austria).

## Results

**Fig. 1:**
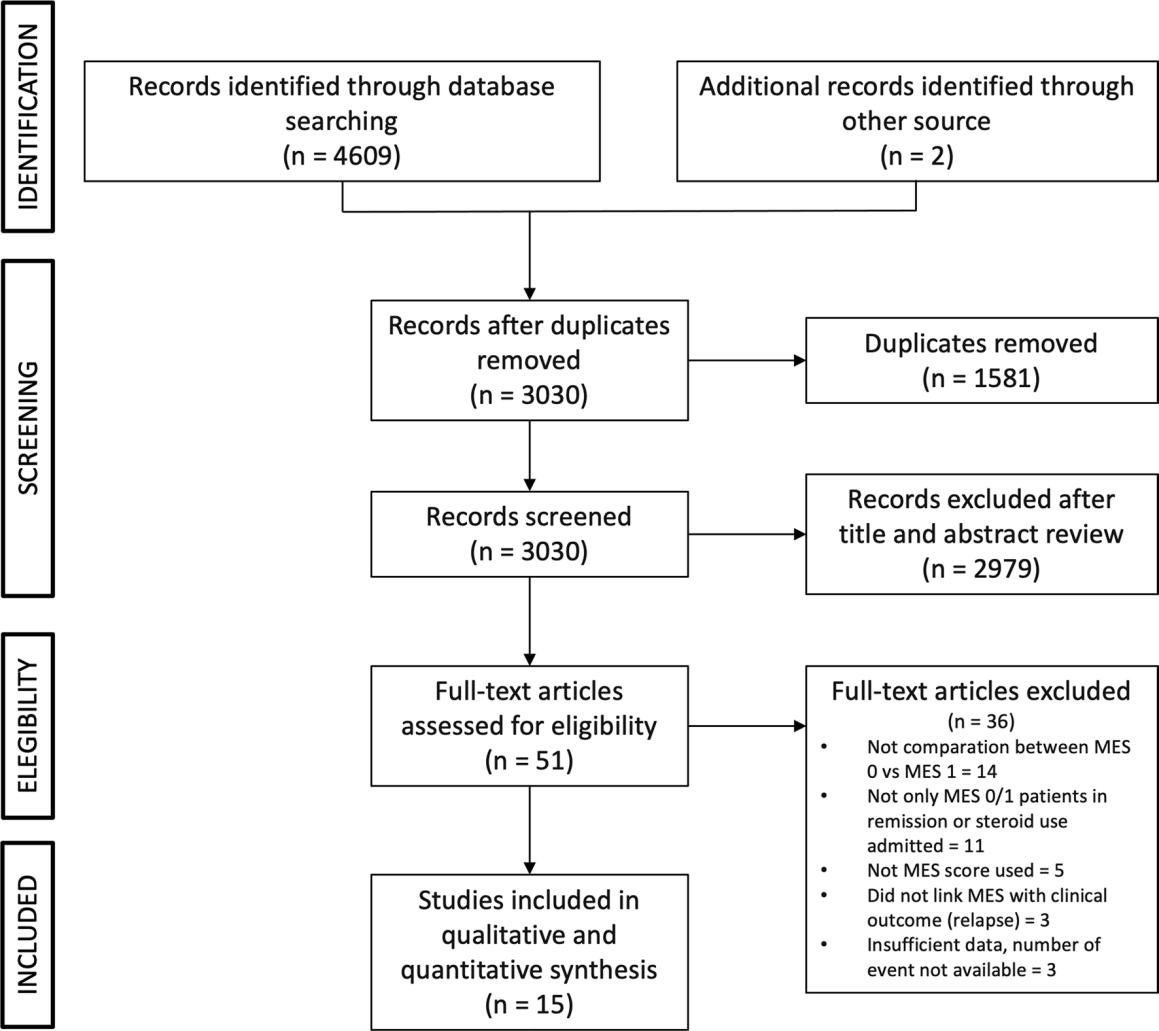
PRISMA flow diagram

Figure 1 shows the PRISMA flow diagram representing the results of the literature search, as assessed by the three authors (MV, AC, and GS). We found 4611 articles, removing 1581 duplicated records, excluding 2979 records based on their titles and abstracts, and 36 based on their full texts evaluation. We included 15 articles for this review. Characteristics of the 15 selected studies are reported in Additional file 1: Table S3. Additional file 1: Table S2 summarizes the definitions of clinical outcome measures set by individual studies. The quality of the included studies assessed by the NOS is summarized in Additional file 1: Table S4. The mean NOS among the 15 studies included was 6.7 (7 high quality; 8 moderate quality).

### Description of excluded studies

Figure 1 Summarizes the selection process of the studies included in this meta-analysis. The reasons for the exclusion of 36 studies are outlined in Additional file 1: Table S5.

### Characteristics of included studies

In assessingclinical relapse, most of the included studies used the clinical Mayo sub-score [27–31] or the need for any treatment escalation [28–30, 32–36]. Of the others, oneused the Truelove and Witts clinical criteria [37], one the Lichtiger Clinical activity Index [38], one the Rachmilewitz Clinical Activity Index [39], and one the Simple Clinical Colitis Activity Index [40]. Finally, two studies evaluated the worsening of stool frequency and/or presence of rectal bleeding [27, 41].

Follow-up lengthsvaried among the studies and details are provided in Table 1.

**Table 1:** Mayo endoscopic score and clinical relapse

	Study design	Follow-up (months)	Population	MES 0 or 1	Clinical relapse	Δ% relapse
Barreiro-de Acosta et al. [32] (Spain)	Prospective	12	187	187 Mayo 0 = 126 (67.3%) Mayo 1 = 61 (32.7%)	49 (26.2%) Mayo 0 = 24 (19.3%) Mayo 1 = 25 (41%)	Δ% = 21.7; *p* < 0.01
Narang et al. [40] (India)	Prospective	12	76	46 Mayo 0 = 36 (78.3%) Mayo 1 = 10 (21.7%)	12 (26.1%) Mayo 0 = 6 (16.7%) Mayo 1 = 6 (60%)	Δ% = 43.3
Ponte et al. [30] (Portugal)	Retrospective	46–72	82	60 Mayo 0 = 32 (53.3%) Mayo 1 = 28 (46.7%)	19 (31.7%) Mayo 0 = 6 (18.8%) Mayo 1 = 13 (46.4%)	Δ% = 27.6; *p* = 0.02
Boal Carvalho et al. [34] (Portugal)	Retrospective	12	138	138 Mayo 0 = 61 (44.2%) Mayo 1 = 77 (55.8%)	28 (20.3%) Mayo 0 = 7 (11.5%) Mayo 1 = 21 (27.3%)	Δ% = 15.8; *p* = 0.022
Yokoyama et al. [35] (Japan)	Retrospective	60	38	24 Mayo 0 = 9 (37.5%) Mayo 1 = 15 (62.5%)	11 (45.8%) Mayo 0 = 2 (22%) Mayo 1 = 9 (60%)	Δ% = 38
Kim et al. [31] (South Korea)	Retrospective	80	215	200 Mayo 0 = 113 (56.5%) Mayo 1 = 87 (43.5%)	51 (25.5%) Mayo 0 = 22 (19.5%) Mayo 1 = 29 (33.3%)	Δ% = 13.8; *p* < 0.023
Yoshino et al. [36] (Japan)	Retrospective	16	298	88 Mayo 0 = 43 (48.9%) Mayo 1 = 45 (51.1%)	21 (23.9%) Mayo 0 = 7 (33.3%) Mayo 1 = 14 (66.7%)	Δ% = 33.4; *p* = 0.167
López-Palacios et al. [37] (Spain)	Prospective	27	20	13 Mayo 0 = 10 (76.9%) Mayo 1 = 3 (23.1%)	2 (15.4%) Mayo 0 = 1 (10%) Mayo 1 = 1 (33.3%)	Δ% = 23.3
Yamamoto et al. [27] (Japan)	Prospective	12	164	164 Mayo 0 = 84 (51%) Mayo 1 = 80 (49%)	46 (28%) Mayo 0 = 19 (22.6%) Mayo 1 = 27 (33.8%)	Δ% = 11.2; *p* = 0.16
Frieri et al. [33] (Italy)	Prospective	36	52	46 Mayo 0 = 29 (63%) Mayo 1 = 17 (37%)	20 (43.5%) Mayo 0 = 9 (31%) Mayo 1 = 11 (64.7%)	Δ% = 33.7; *p* < 0.0001
Lobatón et al. [28] (Belgium, Spain)	Prospective	12	96	96 Mayo 0 = 63 (66%) Mayo 1 = 33 (34%)	22 (23%) Mayo 0 = 13 (21%) Mayo 1 = 9 (27%)	Δ% = 6; *p* = 0.438
Osterman et al. [29] (USA)	Prospective	12	100	61 Mayo 0 = 5 (8.2%) Mayo 1 = 56 (91.8%)	8 (13.1%) Mayo 0 = 0 (0%) Mayo 1 = 8 (14.3%)	Δ% = 14.3
Inoue et al. [41] (Japan)	Retrospective (Abstract)	39	331	254 Mayo 0 = 176 (69%) Mayo 1 = 78 (31%)	53 Mayo 0 = 20 (11.4%) Mayo 1 = 33 (42.3%)	Δ% = 30.9; *p* = 0.017
Kanazawa et al. [39] (Japan)	Retrospective	24	166	166 Mayo 0 = 91 (54.8%) Mayo 1 = 75 (45.2%)	9 Mayo 0 = 3 (3.3%) Mayo 1 = 6 (8%)	Δ% = 4.7
Sakemi et al. [38] (Japan)	Retrospective (Abstract)	36	74	74 Mayo 0 = 23 (31%) Mayo 1 = 51 (69%)	26 Mayo 0 = 2 (9%) Mayo 1 = 23 (46%)	Δ% = 37

Although several studies had 12 months follow-up [27–29, 32, 34, 40], ten studies used a longer period of observation, ranging from 16 to 80 months [30, 31, 33, 35–39, 41]. Seven studies used a prospective design [27–29, 32, 33, 37, 40], while eight studies used a retrospective design [30, 31, 34–36, 38, 39, 41].

Six studies [28, 31–34, 37] assessed the number of patients who underwent colectomy, while three studies [28, 33, 34] reported the hospitalization rate of MES 0 and MES 1 separately (Table 2).

**Table 2:** Mayo endoscopic score, colectomy, and hospitalization

References (Country)	Study design	Follow-up (months)	Population	MES 0 or 1	Colectomy	Hospitalization
Barreiro-de Acosta et al. [32] (Spain)	Prospective	12	187	187 Mayo 0 = 126 (67.3%) Mayo 1 = 61 (32.7%)	0	n.a.^†^
Boal Carvalho et al. [34] (Portugal)	Retrospective	12	138	138 Mayo 0 = 61 (44.2%) Mayo 1 = 77 (55.8%)	0	3 (2.2%) Mayo 0 = 1 (1.6%) Mayo 1 = 2 (2.6%)
Kim et al. [31] (South Korea)	Retrospective	80	215	200 Mayo 0 = 113 (56.5%) Mayo 1 = 87 (43.5%)	0	n.a.^†^
Frieri et al. [33] (Italy)	Prospective	36	52	46 Mayo 0 = 29 (63%) Mayo 1 = 17 (37%)	0	3 (6.5%) Mayo 0 = 0 Mayo 1 = 3 (17.6%)
Lobatón et al. [28] (Belgium, Spain)	Prospective	12	96	96 Mayo 0 = 63 (66%) Mayo 1 = 33 (34%)	0	0
López-Palacios et al. [37] (Spain)	Prospective	27	20	13 Mayo 0 = 10 (76.9%) Mayo 1 = 3 (23.1%)	0	n.a.^†^

^†^n.a: not applicable

### Mayo endoscopic score and risk of clinical relapse

Fifteen studies assessed the clinical relapse [27–41] and included 1617 patients: 901 with MES of 0, and 716 with MES of 1 (Table 1).

**Fig. 2:**
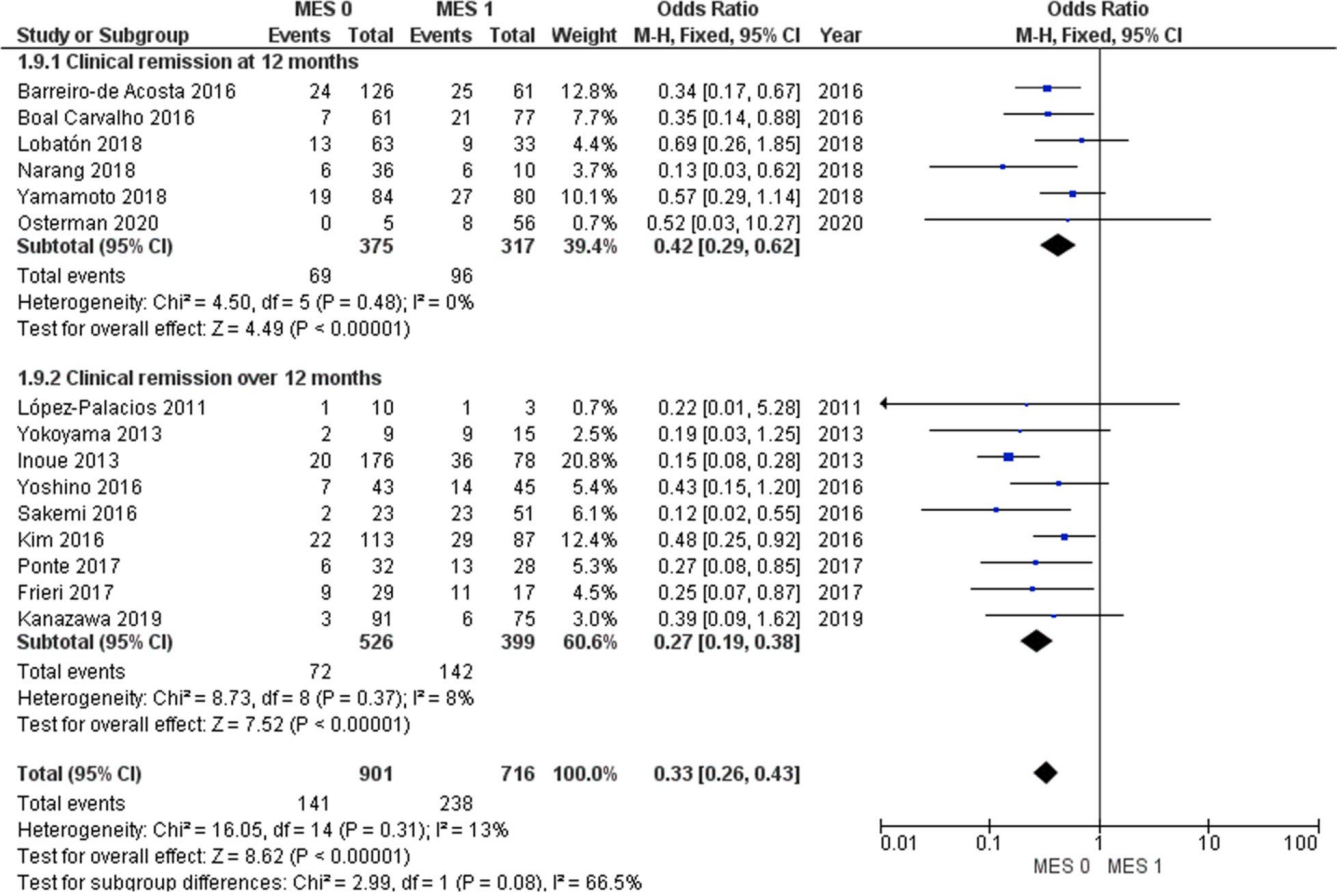
Clinical relapse in MES 0 versus MES 1

The rate of clinical relapse for MES 1 patients ranged from 8 to 66.7%, and for MES 0 patients from 0 to 33.3%. In MES 0 group the pooled odds ratio (OR) for clinical relapse, irrespective of the time of follow-up, was 0.33 (95% CI 0.26–0.43; I^2^ = 13%) (Fig. 2). In particular, the OR was 0.42 (95% CI 0.29–0.62; I^2^ = 0%) in studies with 12 months of follow-up, and 0.27 (95% CI 0.19–0.38, Chi^2^ = 8.73, I^2^ = 8%) in studies with follow-ups longer than 12 months. In the latter subgroup analysis, the median follow-up timewas 27 months (range 16–80) (Fig. 2). Therefore, despite the heterogeneity in the length of follow-up (12–80 months), the subgroup analysis showed that the risk of clinical relapse remained significantly higher in patients with MES 1 compared to MES 0 in studies that followed patients up to 12 months and those that followed patients for longer than 12 months.

**Fig. 3:**
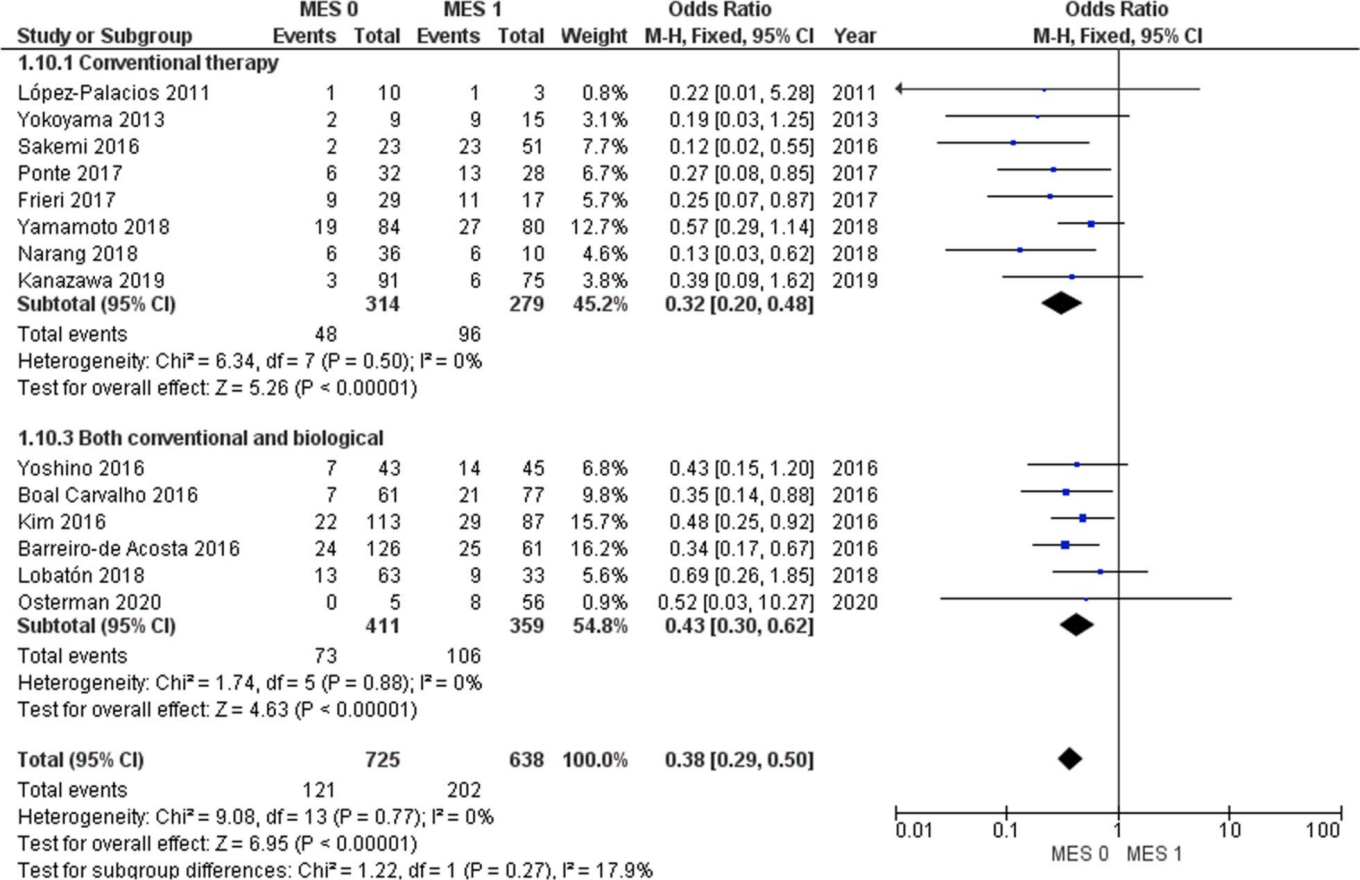
Clinical relapse in MES 0 versus MES 1 regarding therapy

With regard to maintenance therapy, a significantly lower clinical relapse was observed in patients with MES 0 than with those MES 1 in both sub-groups including only patients in conventional therapy and studies including patients on conventional or biological therapy (Fig. 3).

### Mayo endoscopic score and hospitalization rate

Three studies reported hospitalization rate [28, 33, 34], a zero rate being reportedin one of them [28] (Table 2). Hospitalization rate in the group with a MES of 0 ranged from 0 to 1.6%; on the other hand, it was 0–17.6% in MES 1 group.

**Fig. 4:**
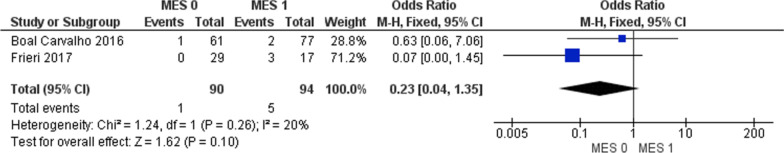
Hospitalization in MES 0 versus MES 1

Patients achieving MES 0 had a lower risk of hospitalization (OR 0.23; 95% CI 0.04–1.35; I^2^ = 20%), although the small numbers involved preclude general significance (Fig. 4).

### Mayo endoscopic score and risk of colectomy

Six studies [28, 31–34, 37] assessed the number of patients undergoing colectomy (Table 2). Among 680 patients no event was recorded for this outcome.

### Sensitivity analysis and publication bias

Sensitivity analysis was conducted excluding the abstracts and the studies with low-moderate quality assessed at NOS. Both analyses showed a lower risk of clinical relapse for the MES 0 group. (OR 0.40; 95% CI 0.30–0.53; I^2^ 0%, and OR 0.34; 95% CI 0.23–0.51; I^2^ 0% respectively) (Additional file 1: Figs. S1 and S2).

After the exclusion of each abstract separately [38, 41], the results were (OR 0.35; 95% CI 0.27–0.45; I^2^ 9%, and OR 0.38; 95% CI 0.29–0.50; I^2^ 0%), respectively (Additional file 1: Figs. S3 and S4).

Furthermore, sensitivity analysis was conducted with studies that included only clinical relapse defined by Partial Mayo Score [28–31]. Among these studies, the MES 0 had a lower risk of clinical relapse compared to MES 1 (OR 0.47; 95% CI 0.29–0.77; I^2^ = 0%) (Additional file 1: Fig. S5).

A lower rate of clinical relapse was also observed in MES 0 group after the exclusion of studies with a retrospective design [27–29, 32, 33, 37, 40] (RR 0.54; 95% CI 0.41–0.70; I^2^ = 0) (Additional file 1: Fig. S6).

The asymmetrical shape of the funnel plot and the Egger’s test showed no publication bias among studies analyzing the risk of clinical relapse (*p* = 0.47) (Additional file 1: Fig. S7).

## Discussion

The results of our meta-analysis demonstrate that achieving MES 0 is linked with a lower clinical relapse rate compared to MES 1 in patients with UC in steroid-free clinical remission. Patients with MES 0 had a 67% lower risk of clinical relapse than those with MES 1. A trend toward a lower risk of hospitalization was also observed, but the small number of patients who experienced these outcomes did not allow making firm conclusions. It is nonetheless noteworthy that 88% of the hospitalization occurred in the group of patients with MES 1 at baseline.

MH was firstly reported in 1951 by Kirsner, who observed, using X-ray and proctoscopic examinations, healing of the colonic lesions in a series of patients with severe UC treated with corticotropin. The disappearance of extensive ulcerations of the colon resulted associated with an improved clinical course [42, 43]. More recently, it has been reported that the absence of healing of rectal lesions in patients with severe UC strongly predicts the need for colectomy [44, 45].

In 2015, the Selecting Therapeutic Targets in Inflammatory Bowel Disease (STRIDE) committee defined the treat-to-target approach, based on tailored adjustments of medical therapy, monitoring objective disease activity—i.e., through endoscopy [46].

This strategy has been demonstrated to be feasible in clinical practice and results in high rates of MH long-term prevention of bowel damage and disease complications (hospitalizations, colectomy, dysplasia/cancer) [47].

An unequivocal definition of MH is necessary in order to apply the treat-to-target approach. To date, there is no validated definition of what constitutes endoscopic remission in UC. The most used tool in both routine practice and clinical trials is the MES, being endoscopic remission often defined as both complete (MES 0) and partial (MES 1).

Recently, a prospective multicenter study showed that high definition (HD) electronic chromoendoscopy can detect subtle mucosal and vascular changes that may reflect histologic remission. The Authors develop a new virtual electronic chromoendoscopy score (PICaSSO score) and show this score strongly correlates with histological scores with a better correlation coefficient than the MES and Ulcerative Colitis Endoscopic Index of Severity score in predicting histological remission [48]. These results could represent a valuable tool for a better definition of MH. Unfortunately, only two articles among the included studies in our meta-analysis mentioned technical specifications of the colonoscope in their methods section. These two studies specified the use of virtual chromoendoscopy [28, 35] and neither mentioned the use of HD definition imaging. Therefore, no pooled analysis concerning the risk of clinical relapse with and without HD-defined MES 0 was possible due to inconsistencies in reported outcomes.

Two meta-analyses [49, 50] assessed the impact of MH on long-term outcomes in patients with UC. The authors of both analyses concluded that MH, defined as both complete and partial healing, is a strong predictor of long-term clinical remission. Complete healing predicted higher rates of clinical remission, but it did not result in significantly more favorable than partial healing for predicting surgeries or hospitalizations.These meta-analyses however included studies which used different endoscopic scores (most of them grouping together complete and partial healing) and study populations differing with regard disease activity and therapies.

To overcome these methodologic inadequacies (bias), in our meta-analysis we considered a homogeneous series of studies including only patients with UC in stable clinical remission. The greater strength of our meta-analysis is the rigorous selection of studies including patients in clinical remission without the use of steroids. Moreover, MH was defined based on MES in all studies distinguishing between complete (MES 0) and partial (MES 1) MH. This makes the results of our meta-analysis directly applicable in clinical practice and avoids potential bias in the evaluation of clinical remission.

In all, MES of 0 was associated with greater benefit compared to MES 1 (OR 0.33; 95% CI 0.26–0.43; I^2^ = 13%). The lower risk of clinical relapse in MES 0 patients has been further demonstrated in a subgroup analysis including studies that followed patients up to 12 months (OR 0.42; 95% CI 0.29–0.62; I^2^ = 0%) and those that followed patients for longer than 12 months (OR 0.27; 95% CI 0.19–0.38; I^2^ = 8%). Moreover, in a sub-analysis including only studies with a prospective design (27–29, 32, 33, 37, 40) a lower rate of clinical relapse was observed in the MES 0 group (RR 0.54; 95% CI 0.41–0.70; I^2^ = 0). Hospitalization rates in the group with the MES 0 ranged from 0 to 1.6%, and from 0 to 17.6% in MES 1 group. Although 5/6 of hospitalization occurred in MES 1 group,the patient numbers involved were too small to permit general conclusions to be drawn from this result. Among the studies that assessed the outcome of colectomy, no event was registered.

However, our meta-analysis has several limitations. First, clinical relapse was defined differently among the included studies, ranging from various clinical indices to the need for steroids or the need for escalation therapy. Second, the quality of studies included in our analysis was acceptable in the NOS, but most studies were lack of comparison of population characteristics between the MES 0 and MES 1 group at baseline.

## Conclusions

Our meta-analysis showed a significantly lower risk of clinical relapse in UC patients with an endoscopic finding of MES 0 compared with MES 1, regardless of follow-up length (12 months or longer). MES 0 should therefore be considered the optimal therapeutic goal as it predicts the best clinical course in patients with UC.

## Supplementary Information

**Additional file 1: Table S1.** Research strategy. **Table S2** Clinical outcomes and measures. **Table S3** Baseline characteristics of the included studies. **Table S4** Quality Assessment of Studies by Newcastle-Ottawa Scale. **Table S5** Full text excluded. **Fig. S1** Sensitivity analysis after abstract exclusion. **Fig. S2** Sensitivity analysis after low-moderate quality studies exclusion. **Fig. S3** Sensitivity analysis after abstract^38^ exclusion. **Fig. S4** Sensitivity analysis after abstract^41^ exclusion. **Fig. S5** Sensitivity analysis including studies with clinical relapse assessed with Partial Mayo Score evaluation. **Fig. S6** Sensitivity analysis after retrospective studies exclusion. **Fig. S7** Funnel plot for clinical relapse (N: 15 studies, 1617 patients); Egger’s test: p = 0.47.

## Data Availability

All data generated or analysed during this study are included in this published article [and its Additional file 1 files].

## Abbreviations

MH: Mucosal healing
UC: Ulcertive colitis
MES: Mayo endoscopic score
NRSI: Non-randomized study of intervention
IBD: Inflammatory bowel disease
NOS: New castle–ottawa scale
OR: Odds ratios
