# Supplementary material for: Systematic review and meta-analysis: the advantage of endoscopic Mayo score 0 over 1 in patients with ulcerative colitis
Source: BMC Gastroenterol. 2022 Mar 3;22:92. doi: 10.1186/s12876-022-02157-5 (PMC8895505; doi:10.1186/s12876-022-02157-5)
Supplement: Supplementary file 1 — Additional file 1: Table S1. Research strategy. Table S2 Clinical outcomes and measures. Table S3 Baseline characteristics of the included studies. Table S4 Quality Assessment of Studies by Newcastle-Ottawa Scale. Table S5 Full text excluded. Fig. S1 Sensitivity analysis after abstract exclusion. Fig. S2 Sensitivity analysis after low-moderate quality studies exclusion. Fig. S3 Sensitivity analysis after abstract38 exclusion. Fig. S4 Sensitivity analysis after abstract41 exclusion. Fig. S5 Sensitivity analysis including studies with clinical relapse assessed with Partial Mayo Score evaluation. Fig. S6 Sensitivity analysis after retrospective studies exclusion. Fig. S7 Funnel plot for clinical relapse (N: 15 studies, 1617 patients); Egger’s test: p = 0.47. [file 12876_2022_2157_MOESM1_ESM.docx]

**ADDITIONAL FILE**

**Additional file 1: Table S1: Research strategy**

| ("colitis, ulcerative"[MeSH Terms] OR "inflammatory bowel disease"[All Fields] OR "ulcerative colitis"[All Fields]) |
| --- |
| **AND** |
| ("mucosal healing"[All Fields] OR "endoscopic remission"[All Fields] OR "endoscopic healing"[All Fields] OR "Mayo endoscopic score"[All Fields] OR "Mayo 0"[All Fields] OR "Mayo 1"[All Fields]) |

| **Additional file 1: Table S2: Clinical Outcomes and Measures** | | | | |  |
| --- | --- | --- | --- | --- | --- |
| **Reference (Country)** | **Study design** |  | **Outcome** |  | **Colonoscope technology** |
|  |  | **Clinical Relapse** | **Colectomy** | **Hospitalization** |  |
| Barreiro-de Acosta  2016  *(Spain)* | Prospective | Recatal bleeding AND  Therapy to induce remission  or any treatment escalation | Yes | No | n.a. |
| Narang 2018  *(India)* | Prospective | Simple Clinical Colitis Activity Index > 2 | No | No | n.a. |
| Ponte 2017  *(Portugal)* | Retrospective | Therapy to induce remission  Partial Mayo score ≥2 | No | No | n.a. |
| Boal Carvalho 2016  (Portugal) | Retrospective | Therapy to induce remission  Any treatment escalation | Yes | Yes | n.a. |
| Yokoyama 2013  (Japan) | Retrospective | Any treatment escalation | No | No | High magnification colonoscope |
| Kim 2016  (South Korea) | Retrospective | Partial Mayo score ≥3 | Yes | No | n.a. |
| Yoshino 2016  (Japan) | Retrospective | Any treatment escalation | No | No | n.a. |
| Lόpez-Palacios 2011  (Spain) | Prospective | Truelove and Witt’s criteria | Yes | No | n.a. |
| Yamamoto 2018  (Japan) | Prospective | Worsening of stool frequency and/or rectal bleeding with MES ≥2 | No | No | n.a. |
| Frieri 2017  (Italy) | Prospective | Treatment escalation (Steroid, Immunomodulator, biological drugs) | Yes | Yes | n.a. |
| Lobatόn 2018  (Belgium, Spain) | Prospective | Partial Mayo score ≥3  Any treatment escalation | Yes | Yes | Chromoendoscopy or narrow-banding imaging |
| Osterman 2020  (USA) | Prospective | Partial Mayo score ≥2  Any treatment escalation | No | No | n.a. |
| Inoue 2013  (Japan) | Retrospective (Abstract) | Recatal bleeding AND increase in bowel movement at two consecutive visits | No | No | n.a. |
| Sakemi 2016  (Japan) | Retrospective (Abstract) | Lichtiger Clinical activity Index (CAI) score ≥5 | No | No | n.a. |
| Kanazawa 2019  (Japan) | Retrospective | Rachmilewitz Clinical Activity Index (RCA)I ≥5 | No | No | n.a. |

| **Additional file 1: Table S3:** **Baseline characteristics of the included studies** | | | | | | |
| --- | --- | --- | --- | --- | --- | --- |
| **Reference**  **(Country)** | **Study design** | **Follow-up**  **(months)** | **Population** | **Baseline therapy** | **MES 0 or 1** | **Outcome** |
| Barreiro-de Acosta  2016  *(Spain)* | Prospective | 12 | 187 | None, Mesalamine, IMM^†^, anti-TNFα^‡^ | 187  *Mayo 0= 126 (67.3%)*  *Mayo 1= 61 (32.7%)* | Clinical relapse, Colectomy |
| Narang 2018  *(India)* | Prospective | 12 | 76 | Mesalamine, IMM^†^ | 46  *Mayo 0= 36 (78.3%)*  *Mayo 1= 10 (21.7%)* | Clinical relapse |
| Ponte 2017  *(Portugal)* | Retrospective | 46-72 | 82 | Mesalamine, IMM^†^ | 60  *Mayo 0= 32 (53.3%)*  *Mayo 1= 28 (46.7%)* | Clinical relapse |
| Boal Carvalho 2016  (Portugal) | Retrospective | 12 | 138 | Mesalamine, IMM^†^, anti-TNFα^‡^ | 138  *Mayo 0= 61 (44.2%)*  *Mayo 1= 77 (55.8%)* | Clinical relapse, Colectomy, Hospitalization |
| Yokoyama 2013  (Japan) | Retrospective | 60 | 38 | None, Mesalamine, IMM^†^ | 24  *Mayo 0= 9 (37.5%)*  *Mayo 1= 15 (62.5%)* | Clinical relapse |
| Kim 2016  (South Korea) | Retrospective | 80 | 215 | Mesalamine, IMM^†^, anti-TNFα^‡^ | 200  *Mayo 0= 113 (56.5%)*  *Mayo 1= 87 (43.5%)* | Clinical relapse, Colectomy |
| Yoshino 2016  (Japan) | Retrospective | 16 | 298 | Mesalamine, IMM^†^, anti-TNFα^‡^, GMAA^§^, none | 88  *Mayo 0= 43 (48.9%)*  *Mayo 1= 45 (51.1%)* | Clinical relapse |
| Lόpez-Palacios 2011  (Spain) | Prospective | 27 | 20 | IMM^†^ | 13  *Mayo 0= 10 (76.9%)*  *Mayo 1= 3 (23.1%)* | Clinical relapse, Colectomy |
| Yamamoto 2018  (Japan) | Prospective | 12 | 164 | Mesalamine | 164  *Mayo 0=84 (51%)*  *Mayo 1= 80 (49%)* | Clinical relapse |
| Frieri 2017  (Italy) | Prospective | 36 | 52 | Mesalamine | 46  *Mayo 0=29 (63%)*  *Mayo 1= 17 (37%)* | Clinical relapse, Colectomy, Hospitalization |
| Lobatόn 2018  (Belgium, Spain) | Prospective | 12 | 96 | Mesalamine, IMM^†^, anti-TNFα^‡^ | 96  *Mayo 0= 63 (66%)*  *Mayo 1= 33 (34%)* | Clinical relapse, Colectomy, Hospitalization |
| Osterman 2020  (USA) | Prospective | 12 | 100 | Mesalamine, IMM^†^, anti-TNFα^‡^ | 61  *Mayo 0= 5 (8.2%)*  *Mayo 1= 56 (91.8%)* | Clinical relapse |
| Inoue 2013  (Japan) | Retrospective  (Abstract) | 39 | 331 | n.a.^¶^ | 254  *Mayo 0= 176 (69%)*  *Mayo 1= 78 (31%)* | Clinical relapse |
| Sakemi 2016  (Japan) | Retrospective (Abstract) | 36 | 74 | Mesalamine, IMM^†^ | 74  *Mayo 0= 23 (31%)*  *Mayo 1= 51 (69%)* | Clinical relapse |
| Kanazawa 2019  (Japan) | Retrospective | 24 | 166 | Mesalamine | 166  *Mayo 0= 91 (%)*  *Mayo 1= 75 (%)* | Clinical relapse |

^†^IMM: Azathioprine or 6-mercaptopurine; ^‡^TNF: tumor necrosis factor; ^§^GMAA: granulocyte monocyte adsorption apheresis.
^¶^n.a.: not available.

| **Additional file 1: Table S4: Quality Assessment of Studies by Newcastle-Ottawa Scale** | | | | | |
| --- | --- | --- | --- | --- | --- |
| **Reference**  **(Country)** | **Study design** |  |  |  |  |
|  |  | **Selection** | **Comparability** | **Outcome** | **Total Score**  **Average** |
| Barreiro-de Acosta  2016 *(Spain)* | Prospective | **** | ** | *** | 9 |
| Narang 2018  *(India)* | Prospective | **** | - | ** | 6 |
| Ponte 2017  *(Portugal)* | Retrospective | **** | - | *** | 7 |
| Boal Carvalho 2016  (Portugal) | Retrospective | **** | ** | *** | 9 |
| Yokoyama 2013  (Japan) | Retrospective | **** | - | *** | 7 |
| Kim 2016  (South Korea) | Retrospective | **** | - | ** | 6 |
| Yoshino 2016  (Japan) | Retrospective | *** | ** | *** | 8 |
| Lόpez-Palacios 2011  (Spain) | Prospective | *** | - | *** | 6 |
| Yamamoto 2018  (Japan) | Prospective | ** | - | *** | 5 |
| Frieri 2017  (Italy) | Prospective | *** | - | *** | 6 |
| Lobatόn 2017  (Belgium, Spain) | Prospective | **** | - | ** | 6 |
| Osterman 2020  (USA) | Prospective | **** | - | *** | 7 |
| Inoue 2013  (Japan) | Retrospective (Abstract) | *** | * | ** | 6 |
| Sakemi 2016  (Japan) | Retrospective (Abstract) | ** | * | ** | 5 |
| Kanazawa 2019  (Japan) | Retrospective | **** | ** | ** | 8 |
|  |  |  |  |  |  |

**NOS CRITERIA:**

**(a) selection (score 0–4)**

- patients in steroid-free clinical remission with MES 0 or MES 1 at baseline (drawn from the same representative UC population);
- MES ascertained in the same manner for those with MES 0 or MES 1.

**(b) comparability (score 0–2)**

- comparability of MES 0 and 1 population characteristics at baseline (percent of pancolitis, smokers, years from diagnosis).

**(c) outcome (score 0–3)
-** defined by clinical relapse evaluated with a clinical score, steroid use, or any treatment escalation;
- colectomy and hospitalization with appropriate follow-up to assess these outcomes (≥12 months);
- adequacy of accounting for subjects.

**Additional file 1: Table S5: Full text excluded**

| **n.** | **Study** | **Reasons** |
| --- | --- | --- |
| 1 | Uchiyama, 2017 | Steroid use |
| 2 | Ikeya, 2015 | Steroid use |
| 3 | Naganuma, 2019 | Steroid use |
| 4 | Fukuda, 2019 | No MES 0 patients |
| 5 | Shi, 2015 | No clinical relapse evaluation |
| 6 | Nakarai, 2016 | Steroid use |
| 7 | Nakarai, 2018 | No MES 1 patients |
| 8 | Pouillon, 2018 | No separate evaluation of MES0/1 |
| 9 | Molander, 2014 | No separate evaluation of MES0/1 |
| 10 | Calafat, 2017 | No separate evaluation of MES0/1 |
| 11 | Arai, 2016 | No MES score used (UCEIS) |
| 12 | Fukaura, 2019 | No separate evaluation of MES0/1 |
| 13 | Theede, 2016 | No clinical relapse evaluation |
| 14 | D’Haenf, 2012 | No separate evaluation of MES0/1 |
| 15 | Rosenberg, 2013 | No separate evaluation of MES0/1 |
| 16 | Langhorst, 2013 | No MES score used |
| 17 | Feagins, 2013 | Histological evaluation only |
| 18 | Miyoshi, 2013 | No separate evaluation of MES0/1 |
| 19 | Prantera, 2009 | No MES score used (UCDAI) |
| 20 | Yamamoto, 2015 | No MES score used (UCDAI) |
| 21 | Bessissow, 2012 | No MES 1 patients |
| 22 | Barreiro-de Acosta, 2009 | Steroid use; No colonoscopic evaluation at baseline |
| 23 | Bortlik, 2016 | No separate evaluation of MES0/1 |
| 24 | Colombel, 2011 | No patients in clinical remission only |
| 25 | Bokemeyer, 2012 | No separate evaluation of MES0/1 |
| 26 | Azad, 2011 | No separate evaluation of MES0/1 |
| 27 | Nakarai, 2014 | Steroid use; No clinical relapse evaluation |
| 28 | Farkas, 2014 | No patients in clinical remission only; No separate evaluation MES 0/1 |
| 29 | Takenaka, 2019 | No clinical relapse evaluation available |
| 30 | Bosuyt, 2017 | No patients in clinical remission only |
| 31 | Ikeya, 2016 | Steroid use |
| 32 | Andreoli, 1993 | No MES score used (Endoscopic grading scale) |
| 33 | Dai, 2014 | Evaluation of clinical relapse after discontinuation of therapy |
| 34 | Ozaki, 2019 | Steroid use |
| 35 | Takahashi, 2016 | No clinical relapse evaluation available |
| 36 | Lahaire, 2013 | Steroid use |

**
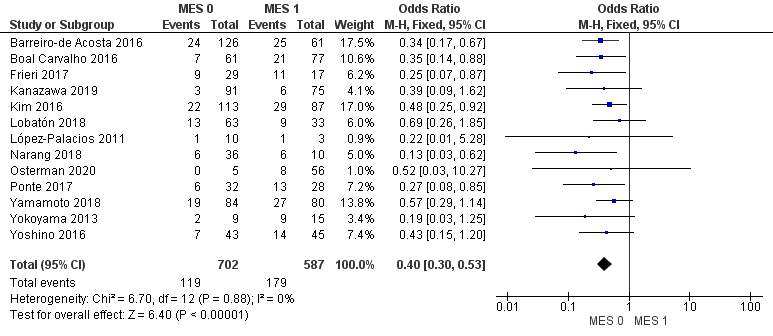
**

**Additional file 1: Figure S1: Sensitivity analysis after abstract exclusion**

**
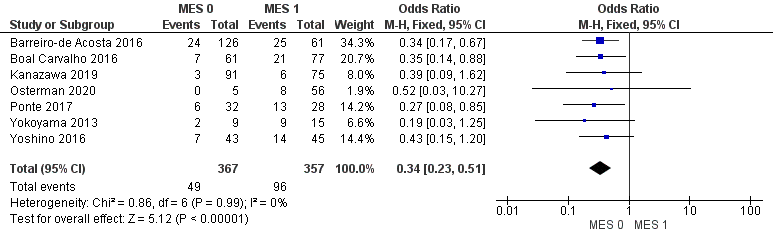
**

**Additional file 1: Figure S2: Sensitivity analysis after low-moderate quality studies exclusion**

**
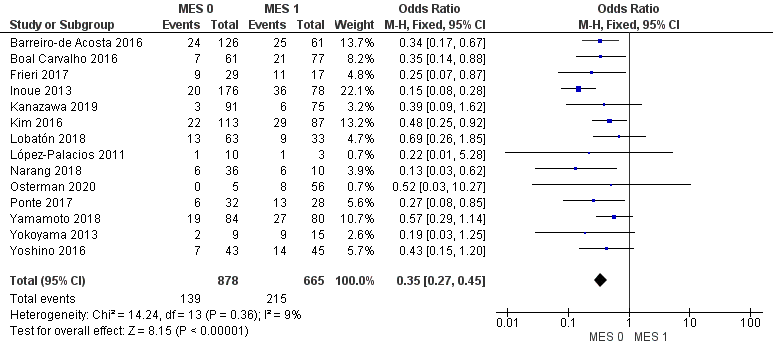
**

**Additional file 1: Figure S3: Sensitivity analysis after abstract^38^ exclusion**

**
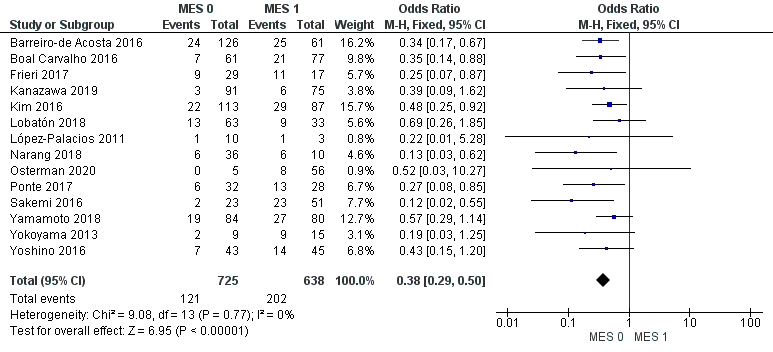
**

**Additional file 1: Figure S4: Sensitivity analysis after abstract^41^ exclusion**

**
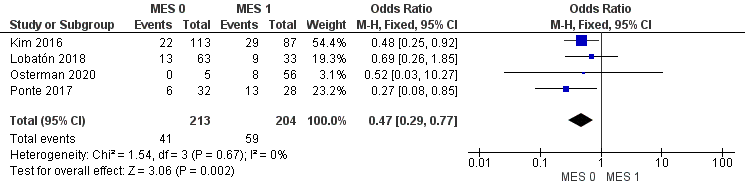
**

**Additional file 1: Figure S5: Sensitivity analysis including studies with clinical relapse assessed with partial Mayo score evaluation**

**
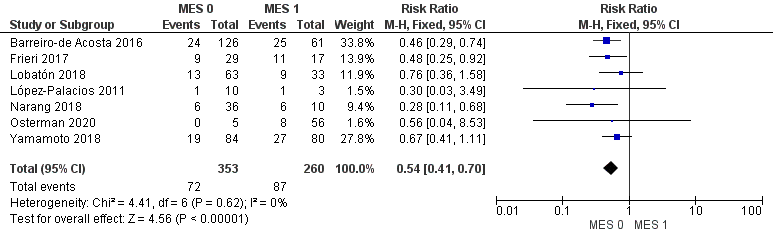
**

**Additional file 1: Figure S6: Sensitivity analysis after retrospective studies exclusion**


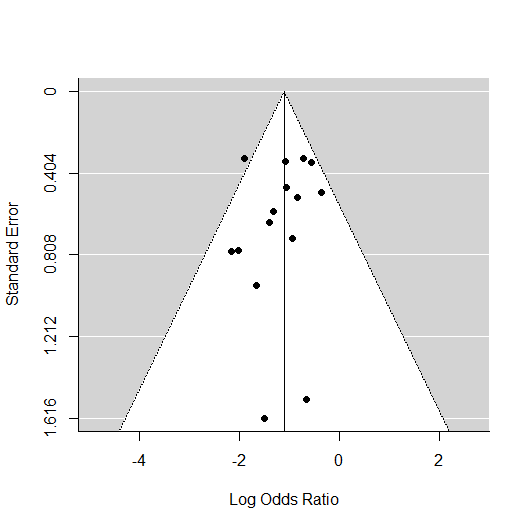


**Additional file 1: Figure S7: Funnel plot for clinical relapse (N: 15 studies, 1617 patients); Egger’s test: p= 0.47**
